# Correlation between structure and temperature in prokaryotic metabolic networks

**DOI:** 10.1186/1471-2105-8-303

**Published:** 2007-08-21

**Authors:** Kazuhiro Takemoto, Jose C Nacher, Tatsuya Akutsu

**Affiliations:** 1 Bioinformatics Center, Institute for Chemical Research, Kyoto University, Gokasho, Uji, Kyoto 611-0011, Japan

## Abstract

**Background:**

In recent years, an extensive characterization of network structures has been made in an effort to elucidate design principles of metabolic networks, providing valuable insights into the functional organization and the evolutionary history of organisms. However, previous analyses have not discussed the effects of environmental factors (i.e., exogenous forces) in shaping network structures. In this work, we investigate the effect of temperature, which is one of the environmental factors that may have contributed to shaping structures of metabolic networks.

**Results:**

For this, we investigate the correlations between several structural properties characterized by graph metrics like the edge density, the degree exponent, the clustering coefficient, and the subgraph concentration in the metabolic networks of 113 prokaryotes and optimal growth temperature. As a result, we find that these structural properties are correlated with the optimal growth temperature. With increasing temperature, the edge density, the clustering coefficient and the subgraph concentration decrease and the degree exponent becomes large.

**Conclusion:**

This result implies that the metabolic networks transit with temperature as follows. The density of chemical reactions becomes low, the connectivity of the networks becomes homogeneous such as random networks and both the network modularity, based on the graph-theoretic clustering coefficient, and the frequency of recurring subgraphs decay. In short, metabolic networks undergo a change from heterogeneous and high-modular structures to homogeneous and low-modular structures, such as random networks, with temperature. This finding may suggest that the temperature plays an important role in the design principles of metabolic networks.

## Background

Elucidation of basic design principles of biological networks is important to understand the cell's internal organization and its adaptation to environmental changes [1]. A large amount of data on molecular interaction networks has recently been accumulated using several new technologies and high-throughput methods. In particular, the increasing number of data on many different organisms has made it possible to trace back the evolutionary history of metabolic networks, and uncover the major structural similarities and differences among species.

The ordinary classification of organisms includes three domains of life [2] (Archaea, Bacteria and Eukaryote) and it is based on cellular organization and similarities among organisms, reflecting common evolutionary history of the species. Archaea and bacteria have a prokaryotic cell organization and are single-celled organisms characterized by the lack of a nucleus cell. In contrast, a eukaryote can be a single-celled or multicellular organism where each cell contains a distinct membrane-bound nucleus. These cells are distinguished from prokaryotic cells by their structural complexity.

However, a different scheme based on the growth temperature ranges can be used to group prokaryote (unicellular) organisms into four classes [3]: Hyperthermophiles (extreme heat-loving), Thermophiles, Mesophiles (grow at moderate temperatures) and Psychrophiles (cold-loving).

On the other hand, the structure of the metabolic networks for many organisms has recently been investigated. For large-scale networks such as metabolic networks, the structural features were analyzed using statistical mechanics and graph theory techniques [4]. In particular, several striking structural properties have recently been found such as small-world [5], scale-free connectivity [6], and hierarchical modularity [7,8] which are absent in random networks [9]. Moreover, sets of ordered substructures such as network motifs [10-12] and highly-interconnected subgraphs [13] that occur far more often than at random have also been detected in different cellular networks. These motifs are thought to be related to specific biological functions [11,12,14], and the recurring pattern in networks reflects the modularity embedded in subcellular systems. These recent findings in network biology suggest that metabolic pathways, as well as the other types of cellular networks, may have been governed by universal laws and basic design principles that might have shaped and optimally organized their structures.

Motivated by the debate on the existence and identification of the design principles, several analyses [15-18] have been carried out. to characterize the structure of metabolic networks, providing important insights into the evolutionary history of metabolism. In Ref. [18], in particular, the authors focused on the three domains of life, and compared the structural properties of the metabolic networks for 11 organisms among their domains of life. As a result, they found that the structural properties of the metabolic networks of bacteria and eukaryote are similar to each other but are quite different when compared with the ones from archaea. However, it is worth noticing that previous analyses were performed using the three domains of life that are defined based on the cellular organization, and consequently they are limited to capture intrinsic structural features of each organism.

One of the possible directions for expanding our knowledge on complex cellular networks is to investigate the effects of exogenous forces induced by environmental conditions on these systems. For example, transcriptional regulatory network is one of the most complex networks in a cell. It is thought that these networks have evolved to optimize the mechanisms responsible for processing external information such as environmental nutrients and a diverse range of stress signals [19]. Then, it is possible that, in general, different environmental conditions may have significantly influenced the structures in cellular networks in a diverse manner.

In this work, we present an analysis for uncovering the effects of exogenous forces in shaping structures in metabolic networks. We used here the KEGG database: Kyoto Encyclopedia of Genes and Genomes [20] and PGTdb: The Prokaryotic Growth Temperature Database [3]. We selected prokaryotes from KEGG whose optimal growth temperature is assigned in PGTdb. We then constructed metabolic networks of the prokaryotes as substrate graphs, in which nodes and edges correspond to metabolites and binary relationships between them, respectively (see Methods for details). In addition, we used two types of the metabolic networks to investigate the effect of ubiquitous metabolites, such as water and ATP, on the correlation between the structural properties and optimal growth temperature. First, we construct a complete network for each prokaryotic organism, which involves all metabolites. Next, a second network for each organism was constructed by deleting 13 nodes as ubiquitous metabolites, which serve for energy exchange, exchange of a proton or a phosphate moiety, and so on, and edges which connect to the ubiquitous metabolites from the complete network (see Methods for details). We then extracted the largest connected components from the complete metabolic networks and selected 113 prokaryotes for each of which the size of the component in the network without ubiquitous metabolites is more than 200. This procedure is done to evaluate more accurately the structural properties.

In 113 prokaryotes, we found 9 hyperthermophiles, 9 thermophiles, 94 mesophiles, and one psychrophile based on PGTdb. Next, we computed several graph metrics to characterize structural properties: the edge density, the degree exponent, the clustering coefficient, and the subgraph concentration corresponding to the metabolic networks of 113 prokaryotes constructed as above. We then investigated the correlation between these structural properties and optimal growth temperature. As a main result, our analysis revealed statistically significant correlations between the network structure and temperature. Although the strength of the correlation is not strong, it is significant enough to suggest that environmental factors play an important role in the design principles of networks.

## Results

### Density of chemical reactions decreases with temperature

First, we show a correlation between the edge density, defined as the ratio of the total number of edges to the total number of nodes, of the metabolic networks and temperature. From biological viewpoint, the edge density represents the ratio of chemical reactions to metabolites. Using the edge density, we can understand how dense the chemical reactions are in metabolic networks.

**Figure 1:**
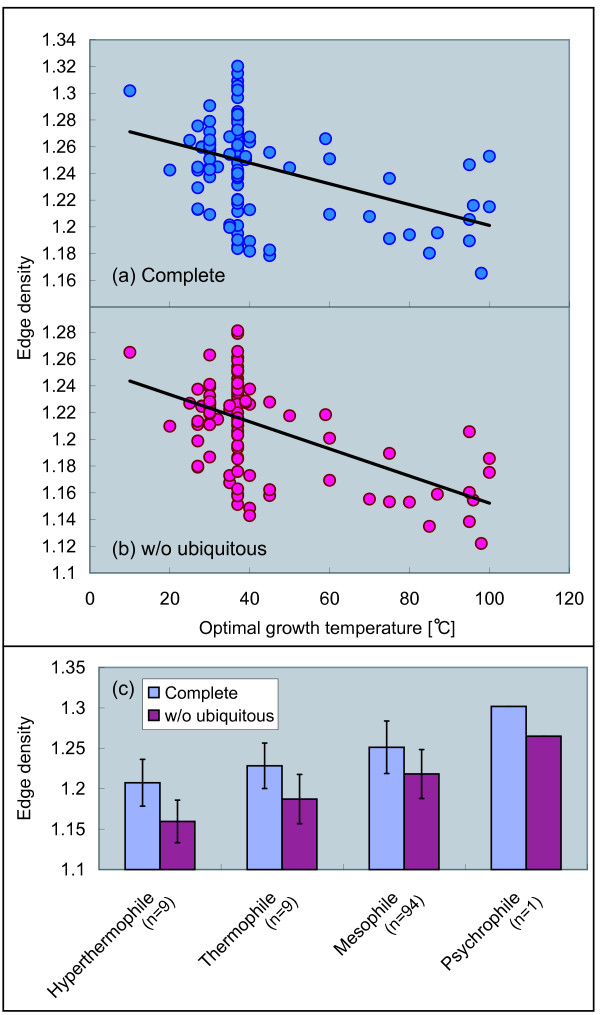
**Correlation between edge density and temperature**. (a) The complete networks (Pearson's correlation *r *= -0.42 with *P *< 10^-5^, Spearman's rank correlation *r*_*s *_= -0.30 with *P *< 0.01), (b) the networks without the ubiquitous metabolite (*r *= -0.54 with *P *< 10^-9^, *r*_*s *_= -0.33 with *P *< 0.001), and (c) averaging based on growth temperature class.

Figures 1(a) and 1(b) show significant negative correlations between the edge density and optimal growth temperature. This feature is observed with both the complete networks and the networks without ubiquitous chemical compounds. As shown in Figure 1, the edge density becomes small with temperature, indicating that density of chemical reactions decreases in metabolic networks with temperature.

### Connectivity becomes homogeneous with temperature

Next, we consider a relationship between the connectivity of the metabolic networks and growth temperature. The connectivity means the number of edges which a metabolites has, and is so-called *degree *in graph theory. In order to characterize the degree, the degree distribution *P*(*k*), defined as the probability that a randomly selected node has exactly *k *edges, is often utilized. Many analyses of metabolic networks using degree distribution have revealed that the degree distribution of metabolic networks follows a power law: *P*(*k*) ∝ *k*^-*γ *^[1,4,6,15,16]. This structural property is called *scale-freeness*. Here, we focus on the degree exponent *γ*, because the exponent reflects a macroscopic tendency of the connectivity in networks. As the degree exponent increases, the probability that a node with large degree exists in a network decreases. That is, most nodes have similar degrees in the networks, indicating that the connectivity of the network is homogeneous as a random network. When the exponent becomes low, in contrast, the probability that a node with large degree exists in a network becomes high. That is, nodes tend to have different degrees in the networks, suggesting that the connectivity of the network is heterogeneous, and therefore is statistically possible to find highly connected nodes or hubs.

**Figure 2:**
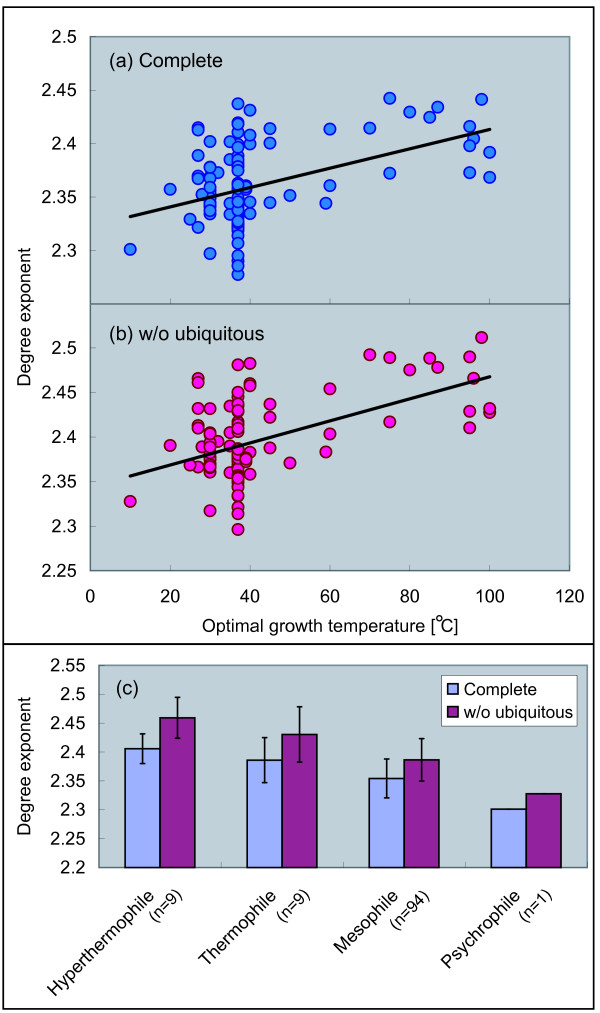
**Correlation between degree exponent and temperature**. (a) The complete networks (Pearson's correlation *r *= 0.45 with *P *< 10^-6^, Spearman's rank correlation *r*_*s *_= 0.27 with *P *< 0.01), (b) the networks without the ubiquitous metabolites (*r *= 0.52 with *P *< 10^-8^, *r*_*s *_= 0.27 with *P *< 0.01), and (c) averaging based on growth temperature class.

Figures 2(a) and 2(b) show significant positive correlations between the degree exponent and optimal growth temperature. This property is conserved between the complete networks and the networks without ubiquitous metabolites. We extracted the degree exponent using the maximum likelihood method (see Methods for details). As shown in Figure 2, the degree exponent increases with temperature, indicating that the connectivity of the metabolic networks becomes homogeneous with temperature.

### Modularity decreases with temperature

Next, we discuss a relationship between the modularity of the metabolic networks and temperature. In this paper, the *modularity *is based on the graph-theoretic clustering coefficient as Ref. [7], and indicates the density of edges among neighbors of a node. And note that the clustering-coefficient-based modularity is different from the edge-betweenness-based modularity [21] and the flux-balance-analysis-based modularity [22]. In this paper, we focus on graph-theoretic aspects, and then the high modularity indicates that reactions among neighboring metabolites of a metabolite are very dense in the metabolic networks. The clustering coefficient is defined as the edge density of the neighbors of a node (see Methods for details), and characterizes the overall tendency of nodes to form clusters in the networks. In the case of high modularity, the clustering coefficient is high.

**Figure 3:**
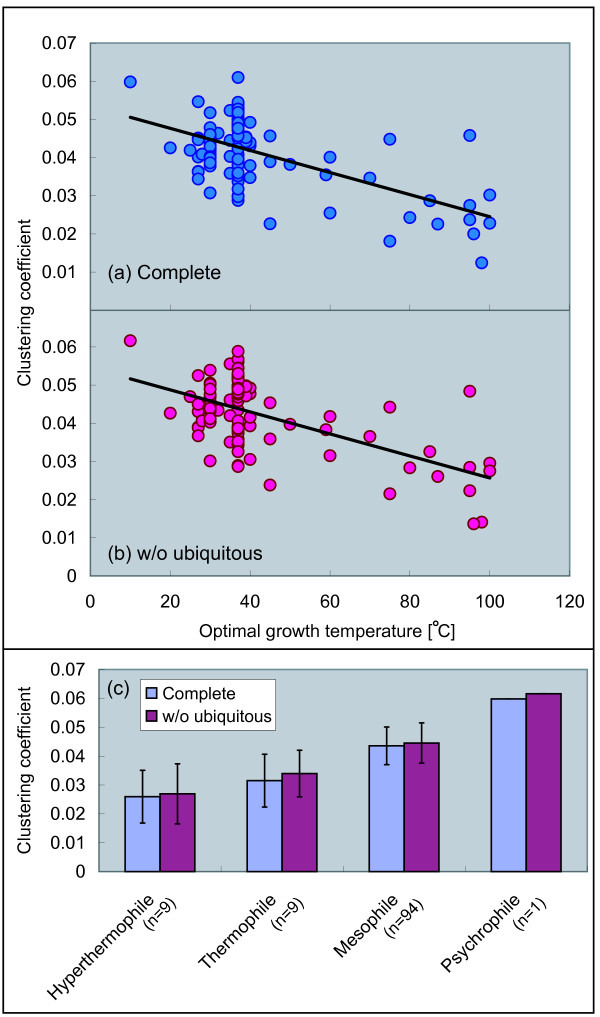
**Correlation between clustering coefficient and temperature**. (a) The complete networks (Pearson's correlation *r *= -0.59 with *P *< 10^-11^, Spearman's rank correlation *r*_*s *_- 0.28 with *P *< 0.01), (b) the networks without the ubiquitous metabolites (*r *= -0.58 with *P *< 10^-10^, *r*_*s *_= -0.30 with *P *< 0.01), and (c) averaging based on growth temperature class.

Figures 3(a) and 3(b) show significant negative correlations between the clustering coefficient and optimal growth temperature. This phenomenon is identical between the complete networks and the networks without ubiquitous metabolites. As shown in Figure 3, the clustering coefficient decays with temperature, implying that the modularity of the networks decreases with temperature.

So far, we have discussed the overall tendency of the modularity in the metabolic networks. Next, we consider a dependency of degree (the number of reactions) on the clustering coefficient to reveal a local tendency of the modularity in the networks. For this, we utilize the degree-dependent clustering coefficient *C*(*k*), defined as the clustering coefficient with degree *k *(see Methods for details).

**Figure 4:**
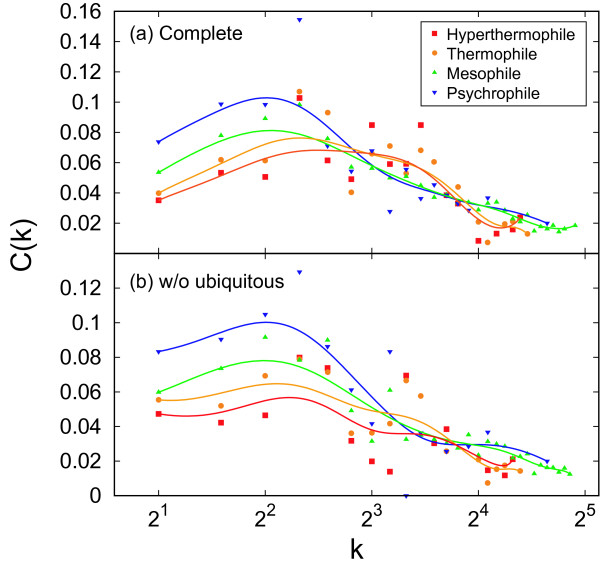
**Degree-dependent clustering coefficient *C*(*k*) for growth temperature class**. (a) The complete networks and (b) the networks without the ubiquitous metabolites. The solid lines are drawn by Bezier curve.

Figures 4(a) and 4(b) show the degree-dependent clustering coefficient *C*(*k*) for the four growth temperature classes. In addition, *C*(*k*) is averaged based on the growth temperature class. As shown in Figure 4, the degree-dependent clustering coefficient tends to decay within small degree (2 ≤ *k *≤ 4) with temperature. For the large degree (*k *≥ 5), in contrast, the clustering coefficient is almost constant even though the temperature changes. This tendency is the same both in complete networks and in the networks without ubiquitous metabolites. In order to evaluate the significance of this variance, we investigated a correlation between the clustering coefficient with degree *k *and optimal growth temperature [see Additional file 1]. Because of space limitation, we do not include additional figures. Instead of the figures, we show the correlation coefficient and the *P*-value for the correlation between *C*(*k*) and optimal growth temperature (Table 1). As shown in Table 1, there tends to be the significant correlations within the small degree. On the other hand, weak correlations are observed for the large degree.

**Table 1:** Correlation coefficient and the *P*-value for the correlation of *C*(*k*) and optimal growth temperature

Network	Complete		w/o ubiquitous metabolites	
*C*(*k*)	*r*	*r*_*s*_	*r*	*r*_*s*_

C(2)	-0.40 (*P *< 10^-5^)	-0.30 (*P *< 0.01)	-0.23 (*P *< 0.05)	-0.12 (*P *= 0.20)
*C*(3)	-0.39 (*P *< 10^-4^)	-0.11 (*P *= 0.23)	-0.55 (*P *< 10^-9^)	-0.31 (*P *< 0.001)
*C*(4)	-0.50 (*P *< 10^-7^)	-0.34 (*P *< 0.001)	-0.52 (*P *< 10^-8^)	-0.33 (*P *< 0.001)
C(5)	0.13 (*P *= 0.17)	0.17 (*P *= 0.07)	-0.27 (*P *< 0.01)	-0.16 (*P *= 0.09)
C(6)	0.04 (*P *= 0.66)	0.15 (*P *= 0.21)	-0.38 (*P *< 10^-4^)	-0.24 (*P *< 0.05)

*r *and *r*_*s *_correspond to Pearson's correlation coefficient and Spearman's rank correlation coefficient, respectively.

This result indicates that the variance of the overall modularity as shown in Figure 3 is caused by the change of the modularity for the metabolites with few chemical reactions in the networks.

### Frequency of appearance of recurring subgraphs decays with temperature

Finally, we argue a relationship between a frequency of appearance of specific subgraphs and temperature. In order to investigate the frequency, we consider (*nt*)-subgraph concentration *C*_*nt *_(see Methods for details). It is well-known that the abundance of recurring subgraphs in biological networks, such as gene regulatory network and metabolic networks, plays an important role in a functional level. These subgraphs are so-called network motifs. In particular, triangles and more complex designs composed of the multiple triangles, occur quite frequently in networks, and tend to be involved in control mechanisms of biological systems [11,12,14]. Since it is believed that the triangles and several larger subgraphs are important in biological networks as above, we focus on two types of the recurring subgraphs: (31)-subgraph (triangle) and (42)-subgraph (square including two triangles), and investigate the subgraph concentrations.

**Figure 5:**
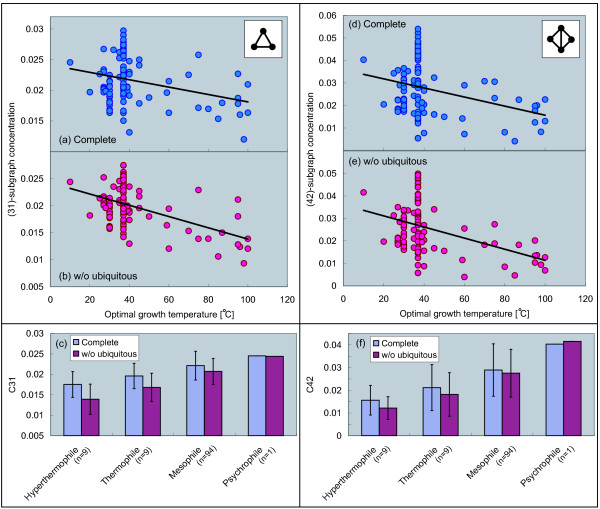
**Correlation between subgraph concentration and temperature**. (31)-subgraph concentration: (a) the complete networks (Pearson's correlation *r *= -0.30 with *P *< 0.01, Spearman's rank correlation *r*_*s *_= -0.10 with *P *= 0.29), (b) the networks without the ubiquitous metabolites (*r *= -0.50 with *P *< 10^-7^, *r*_*s *_= -0.26 with *P *< 0.01), and (c) averaging based on growth temperature class. (42)-subgraph concentration: (d) the complete networks (*r *= -0.32 with *P *< 0.001, *r*_*s *_= -0.21 with *P *< 0.05), (e) the networks without the ubiquitous metabolites (*r *= -0.41 with *P *< 10^-5^, *r*_*s *_= -0.28 with *P *< 0.01), and (f) averaging based on growth temperature class.

Figures 5(b) and 5(e) show significant negative correlations between (31)-subgraph and (42)-subgraph concentration and optimal growth temperature in the networks without ubiquitous metabolites. As shown in Figures 5(a) and 5(c), on the other hand, the correlation in the complete metabolic networks is weak. That is, the correlation becomes clear after removal of ubiquitous metabolites. As shown in Figure 5, the subgraph concentration of the networks without ubiquitous metabolites decays with temperature, suggesting that the probability to find the analyzed subgraphs in the networks decreases with temperature.

## Discussion

From this analysis, several correlations between structural properties and temperature have been revealed. In particular, we have found that the decrease in the edge density, the clustering coefficient and the subgraph concentration and the increase in the degree exponent were correlated with increasing temperature. In the following, we briefly discuss several interesting issues related to our results.

As shown in Figure 5, the correlation between structure and temperature becomes more significant (see correlation coefficient and P-value) in the case of removing ubiquitous metabolites. This implies that the correlations between structure and temperature are independent of the ubiquitous metabolites and the reactions in which the metabolites are involved. That is, it is expected that there are relevant temperature-dependent chemical reactions in metabolic networks. We may be able to find such reactions via a comparison of metabolic networks between in thermophiles and in non-thermophiles.

Now, we speculate how the structural properties of metabolic networks changes with temperature. Structure of networks is determined by connections among nodes. We then contemplate that enzymes that catalyze chemical reactions are concerned in the structural transition of metabolic networks with increasing temperature.

First, we discuss the variance of the density of the chemical reactions with temperature. The density of the chemical reactions becomes low with temperature. One hypothesis could be as follows. It is believed that metabolic networks evolve by gene duplication [24,25]. That is, the number of chemical reactions increases when new enzymes generated by gene duplication stay and act in metabolic networks. In addition, it is reported that the selective constraint at the amino acid level in thermophiles is stronger than that in nonthermophiles [26]. This implies that it is hard for the new enzymes generated by gene duplication to stay in metabolic networks of thermophiles. For this reason, therefore, we speculate that the density of chemical reactions of hyperthermophile and thermophiles is lower than that of mesophile and psychrophiles.

Second, we speculate about the change of the clustering coefficient and subgraph concentration with temperature. The clustering coefficient and the subgraph concentration decay with temperature. One possible explanation is as follows. As before, it is believed that the evolution of metabolic networks is caused by gene duplication. A new enzyme generated by gene duplication tends to be functionally similar to the original enzyme. That is, we expect that the new enzyme tends to catalyze chemical reactions among neighbors of the metabolites in the chemical reaction catalyzed by the original enzyme, when the new enzyme stays and acts in metabolic networks. By gene duplication, as a result, it is conjectured that chemical reactions among the neighbor metabolites of a metabolite are dense, and triangles are generated.

As above, it may be hard for the new enzymes generated by gene duplication to stay in metabolic networks of thermophiles because of the strong selective constraint. Therefore, connections among neighbors of a metabolite and generation of triangles may be inhibited with increasing temperature. For this reason, the clustering coefficient and the subgraph concentration may decay with temperature.

We consider the variance of the connectivity with the temperature and speculate about its origin. The degree exponent increases with temperature, indicating that connectivity becomes homogeneous with temperature. It is well-known that the degree distribution of metabolic networks follows a power law [1,4,6,15,16]. It is important for emergence of the power law to consider a preferential attachment mechanism which means that metabolites with many reactions better tend to get new reaction [1,4,6]. As a result, connectivity is heterogeneous due to the preferential attachment. Actually, it have been confirmed that the metabolic networks growth through the preferential attachment [27]. That is, it is expected that the heterogeneity of connectivity becomes strong when the number of chemical reactions increases. In addition, it is believed that the preferential attachment is induced by gene duplication and gene mutation [28]. As above, the tendency of increment of chemical reactions becomes weak with temperature because of the strong selective constraint. Therefore, connectivity in hyperthermophilic and thermophilic metabolic networks may be more homogeneous than that in mesophilic and psychrophilic metabolic networks.

Moreover, our results can provide new insights into recent analyses on metabolic networks. In Ref. [18], it was shown that the metabolic networks of archaea are essentially different from the metabolic networks of bacteria. Most archaea tend to belong to hyperthermophiles and thermophiles, and most bacteria tend to be referred to mesophiles. We have shown here that the network structure is different among growth temperature classes. Thus, it may suggest that network structures are different between archaea and bacteria because they belong to different growth temperature classes.

**Figure 6:**
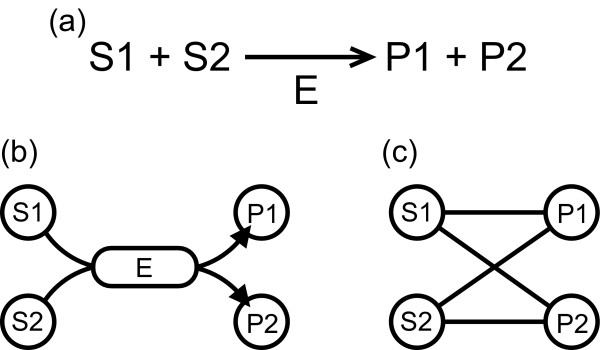
**Representations of metabolic networks**. (a) A chemical reaction catalyzed by an enzyme E. (b) The representation of the reaction (a) in KEGG. (c) The representation of (a) in the metabolic network we used.

In this work, the metabolic networks are represented as simple graphs and are described by binary relationships between substrates and products as shown in Figure 6. This graph representation has been widely used in a series of works exploring the topology of metabolic networks [7,15]. However, it is worth noticing that this representation does not consider all metabolic information, as for example the reaction directions and the stoichiometric matrix (S-matrix). In addition, some works have focused on exploring metabolic networks with different methods and techniques such as triad significance profile [29], the metabolic control analysis [30], the elementary mode analysis [31]. Among the recent topological studies of metabolic networks, the topological analysis of an S-graph constructed from the S-matrix was recently suggested in [32]. The S-graph is represented as a complicated graph with two types of nodes: reactions nodes and metabolite nodes. In this work, in contrast, we consider simple graphs represented with one type of nodes as [1,7,15], which in some cases may suppress relevant biochemical information. The topological analysis based on the S-matrix also allows us to visualize the complete pathways from basic chemical precursors to complex synthesized molecules. Thus, this representation is useful to investigate biochemical mechanisms behind metabolic networks. However, we believe that our graph representation is suitable for the aim of the present study because it is hard to analyze several structural properties of the complicated graphs due to that the development of tools for analysis of the complicated graphs has been yet more backward than that of simple graphs.

On the other hand, we have not included eukaryotic metabolic networks which are absent in the growth temperature database: PGTdb [3]. However, most eukaryote (e.g. plants and animals) belongs to mesophiles because the eukaryote grows at normal temperature (≈ 37°C). Therefore, we predict that structures of metabolic networks are similar between eukaryote and bacteria, which also belong to mesophiles. In Ref. [18], it was recently reported that the network structures are similar between the bacteria and the eukaryote, therefore our results are in agreement with this previous study.

To conclude this section, we note that most of the earliest theories for evolving networks [4,33] have not considered the effect of external factors. This is particularly true for the case of the temperature, which is one of the important environmental factors. Therefore, the establishment of a complete theory for the network evolution considering such environmental factors is an important future challenge.

## Conclusion

We have explored here the relationship between structure and temperature, which is one of environmental forces, in metabolic networks of prokaryotic organisms. As a result, we have found statistically significant correlations between structure and temperature. However, it should be noted that this correlation is not strong. Our findings indicate that the structure of metabolic networks tends to transit with temperature as follows. The density of chemical reactions becomes low, the connectivity of the networks becomes homogeneous such as random networks, the clustering-coefficient-based modularity of the networks becomes small, and the frequency of recurring subgraphs decays. In short, this result implies that metabolic networks transit from heterogeneous and high-modular (high-clustered) structures to homogeneous and low-modular (less-clustered) structures, such as random networks, with temperature. This finding raises the question of what mechanisms were developed during the evolution that induced the absence (or presence) of specific enzymes (chemical reactions) in response to different temperature. Furthermore, our findings also suggest that another possible exogenous force may exist which could also be responsible for shaping biological networks. Therefore, further studies in this research line may provide valuable insights into the evolution of networks.

## Methods

### Network construction

We downloaded the metabolic pathways of 113 prokaryotes from KEGG: Kyoto Encyclopedia of Genes and Genomes [20]. In the metabolic pathways, a chemical reaction catalyzed by an enzyme, e.g. Figure 6(a), is expressed as a directed bipartite graph that consists of enzymes and metabolites by consideration of connections between substrates and products without stoichiometry as shown in Figure 6(b). Here, we expressed the metabolic pathways as a undirected graph as shown in Figure 6(c), by assuming the metabolites and the binary relationship between them as nodes and unweighted and undirected edges, respectively. That is, the metabolic networks only describe a binary relationship between a substrate and a product. In addition, such graph is so-called a *substrate graph*. For example, we consider a reaction as shown in Figure 6(a). In this reaction, P1 and P2 are produced by S1 and S2 via E. Then, we express this reaction as a directed graph in which each substrate (S1 and S2) connects to E. and E connects to P1 and P2, as shown in Figure 6(b). Here we only focus on the relationship between substrates and products. S1 and S2 are related in the production of P1 and P2. Thus, we express this reaction as a undirected graph in which S1 and S2 connect to P1 and P2, as shown in Figure 6(c).

For the metabolic networks, we considered two situations: one is the case of including all metabolites, and another is the case of removing 13 ubiquitous metabolites: water, ATP, ADP, NAD, NADH, NADPH, carbon dioxide, ammonia, sulfate, thioredoxin, (ortho) phosphate (P), pyrophosphate (PP), and H^+^. In the case of the removing 13 ubiquitous metabolites, the metabolic networks are constructed as follows. In Figure 6(b), for example, if S1 is a ubiquitous metabolite, then we remove node S1 and the link between node S1 and node E. And we transform the bipartite graph generated by this procedure into a substrate graph represented by the metabolites and the binary relationship between them as shown in Figure 6(c). As it is known that in the case of removing the ubiquitous metabolites, the network structure may undergo drastic changes [15-17].

### Optimal growth temperature and growth temperature class

We got optimal growth temperature of 113 prokaryotes from PGTdb: The Prokaryotic Growth Temperature Database [3]. And, we classified the prokaryotes into four growth temperature classes according to PGTdb.

### Maximum likelihood estimate of degree exponent

Assuming that degree distribution of the metabolic networks follows a power law: *P*(*k*) ∝ *k*^-*γ*^, the degree exponents *γ *are extracted using maximum likelihood estimate given by the formula [23]

γ=1+N[∑i=1Nln⁡kikmin]−1,
 MathType@MTEF@5@5@+=feaafiart1ev1aaatCvAUfKttLearuWrP9MDH5MBPbIqV92AaeXatLxBI9gBaebbnrfifHhDYfgasaacH8akY=wiFfYdH8Gipec8Eeeu0xXdbba9frFj0=OqFfea0dXdd9vqai=hGuQ8kuc9pgc9s8qqaq=dirpe0xb9q8qiLsFr0=vr0=vr0dc8meaabaqaciaacaGaaeqabaqabeGadaaakeaaiiGacqWFZoWzcqGH9aqpcqaIXaqmcqGHRaWkcqWGobGtdaWadaqaamaaqahabaGagiiBaWMaeiOBa4galeaacqWGPbqAcqGH9aqpcqaIXaqmaeaacqWGobGta0GaeyyeIuoakmaalaaabaGaem4AaS2aaSbaaSqaaiabdMgaPbqabaaakeaacqWGRbWAdaWgaaWcbaGaemyBa0MaemyAaKMaemOBa4gabeaaaaaakiaawUfacaGLDbaadaahaaWcbeqaaiabgkHiTiabigdaXaaakiabcYcaSaaa@497D@

where *N *corresponds to the number of nodes in the metabolic networks, and *k*_*i *_is degree (number of edges) of node *i*. *k*_*min *_is the smallest degree in the networks.

### Clustering coefficient

The clustering coefficient of node *i *with degree *k*_*i *_is defined as *C*_*i *_= 2*M*_*i*_/[*k*_*i*_(*k*_*i *_- 1)] [5,7], where *M*_*i *_denotes the number of edges among neighbors of node *i*. Moreover, we define the average value of *C*_*i *_as the clustering coefficient:

〈C〉=1N∑i=1NCi.
 MathType@MTEF@5@5@+=feaafiart1ev1aaatCvAUfKttLearuWrP9MDH5MBPbIqV92AaeXatLxBI9gBaebbnrfifHhDYfgasaacH8akY=wiFfYdH8Gipec8Eeeu0xXdbba9frFj0=OqFfea0dXdd9vqai=hGuQ8kuc9pgc9s8qqaq=dirpe0xb9q8qiLsFr0=vr0=vr0dc8meaabaqaciaacaGaaeqabaqabeGadaaakeaacqGHPms4cqWGdbWqcqGHQms8cqGH9aqpdaWcaaqaaiabigdaXaqaaiabd6eaobaadaaeWbqaaiabdoeadnaaBaaaleaacqWGPbqAaeqaaaqaaiabdMgaPjabg2da9iabigdaXaqaaiabd6eaobqdcqGHris5aOGaeiOla4caaa@3E9A@

### Degree-dependent clustering coefficient

The degree-dependent clustering coefficient means the average clustering coefficient of nodes with degree *k*, and is defined as

C(k)=∑i=1NCi×δ(ki−k)∑i=1Nδ(ki−k),
 MathType@MTEF@5@5@+=feaafiart1ev1aaatCvAUfKttLearuWrP9MDH5MBPbIqV92AaeXatLxBI9gBaebbnrfifHhDYfgasaacH8akY=wiFfYdH8Gipec8Eeeu0xXdbba9frFj0=OqFfea0dXdd9vqai=hGuQ8kuc9pgc9s8qqaq=dirpe0xb9q8qiLsFr0=vr0=vr0dc8meaabaqaciaacaGaaeqabaqabeGadaaakeaacqWGdbWqcqGGOaakcqWGRbWAcqGGPaqkcqGH9aqpdaWcaaqaamaaqadabaGaem4qam0aaSbaaSqaaiabdMgaPbqabaGccqGHxdaTiiGacqWF0oazcqGGOaakcqWGRbWAdaWgaaWcbaGaemyAaKgabeaakiabgkHiTiabdUgaRjabcMcaPaWcbaGaemyAaKMaeyypa0JaeGymaedabaGaemOta4eaniabggHiLdaakeaadaaeWaqaaiab=r7aKjabcIcaOiabdUgaRnaaBaaaleaacqWGPbqAaeqaaOGaeyOeI0Iaem4AaSMaeiykaKcaleaacqWGPbqAcqGH9aqpcqaIXaqmaeaacqWGobGta0GaeyyeIuoaaaGccqGGSaalaaa@55A5@

where *δ*(*x*) is Kronecker's delta function.

### Subgraph and the concentration

**Figure 7:**
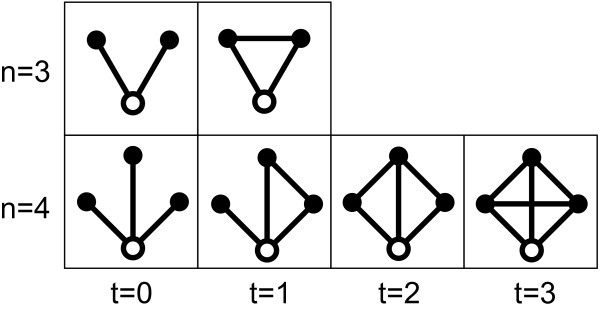
**Types of 3 and 4-node subgraphs**. (*nt*)-subgraph with *n *nodes and *n *- 1 + *t *edges whose central node (the white nodes) has edges to *n *- 1 neighbors, which in turn have *t *links among themselves.

The (*nt*)-subgraph consists of a central node, *n *- 1 neighbors and *n *- 1 + *t *edges, where *t *denotes the number of edges among the neighbors [13]. That is, a subgraph composed of *n *nodes contains (*n *- 1)(*n *- 2)/2 + 1 different subgraphs because the maximal value of *t *is (n−12)
 MathType@MTEF@5@5@+=feaafiart1ev1aaatCvAUfKttLearuWrP9MDH5MBPbIqV92AaeXatLxBI9gBaebbnrfifHhDYfgasaacH8akY=wiFfYdH8Gipec8Eeeu0xXdbba9frFj0=OqFfea0dXdd9vqai=hGuQ8kuc9pgc9s8qqaq=dirpe0xb9q8qiLsFr0=vr0=vr0dc8meaabaqaciaacaGaaeqabaqabeGadaaakeaadaqadaqaauaabeqaceaaaeaacqWGUbGBcqGHsislcqaIXaqmaeaacqaIYaGmaaaacaGLOaGaayzkaaaaaa@3276@. Figure 7 shows the types of 3 and 4 node-subgraphs. The subgraphs are more interconnected with increasing *t*.

The subgraph concentration *C*_*nt *_[13] denotes a fraction of (*nt*)-subgraph abundance in all types of *n*-node subgraphs, and is defined as

Cnt=Snt∑i=0(n−1)(n−2)/2Sni,
 MathType@MTEF@5@5@+=feaafiart1ev1aaatCvAUfKttLearuWrP9MDH5MBPbIqV92AaeXatLxBI9gBaebbnrfifHhDYfgasaacH8akY=wiFfYdH8Gipec8Eeeu0xXdbba9frFj0=OqFfea0dXdd9vqai=hGuQ8kuc9pgc9s8qqaq=dirpe0xb9q8qiLsFr0=vr0=vr0dc8meaabaqaciaacaGaaeqabaqabeGadaaakeaacqWGdbWqdaWgaaWcbaGaemOBa4MaemiDaqhabeaakiabg2da9maalaaabaGaem4uam1aaSbaaSqaaiabd6gaUjabdsha0bqabaaakeaadaaeWaqaaiabdofatnaaBaaaleaacqWGUbGBcqWGPbqAaeqaaaqaaiabdMgaPjabg2da9iabicdaWaqaaiabcIcaOiabd6gaUjabgkHiTiabigdaXiabcMcaPiabcIcaOiabd6gaUjabgkHiTiabikdaYiabcMcaPiabc+caViabikdaYaqdcqGHris5aaaakiabcYcaSaaa@4C25@

where *S*_*nt *_corresponds to (*nt*)-subgraph abundance.

### Statistical analysis

In order to assess the significance of the observed correlations, we used Pearson's correlation coefficient *r*, Spearman's rank correlation coefficient *r*_*s *_and, their *P*-value *P*. We determine that there is a significant correlation between the structural property and optimal growth temperature when *P *< 0.05.

## Authors' contributions

KT and JCN conceived and designed the study. KT analyzed the data and drafted the manuscript. TA provided valuable discussions and suggestions during the development of the manuscript. KT, JCN, and TA read and approved the final manuscript.

## Supplementary Material

Additional file 1 Structural properties and optimal growth temperature of 113 prokaryotes. This file includes the structural properties of the metabolic network, the optimal growth temperature, the temperature class, and the domain for each organism. Name of the organism is written according to KEGG (see [34] for full name). "Domain" colunm indicates the type of domain of each organism (A: archea, B: bacterium). "Temperature class" column represents the temperature class of each organism (HT: hyperthermophile. T: thermophile, M: mesophile, P: psychrophile). The number of nodes, the number of edges, and all structural properties are obtained from the largest connected component of the metabolic network represented as a substrate graph. Click here for file
